# Supplementary material for: Splice-Junction-Based Mapping of Alternative Isoforms in the Human Proteome
Source: Cell Rep. Author manuscript; Available in PMC 2020 Jan 15. (PMC6961840; doi:10.1016/j.celrep.2019.11.026)
Supplement: 2 [file NIHMS1546469-supplement-2.zip › DF1/PXD010154/Spleen/Spleen_6_SRSF11_VTAQPDVLEVQAEYITAGPGPSGGPGGGG.pdf]

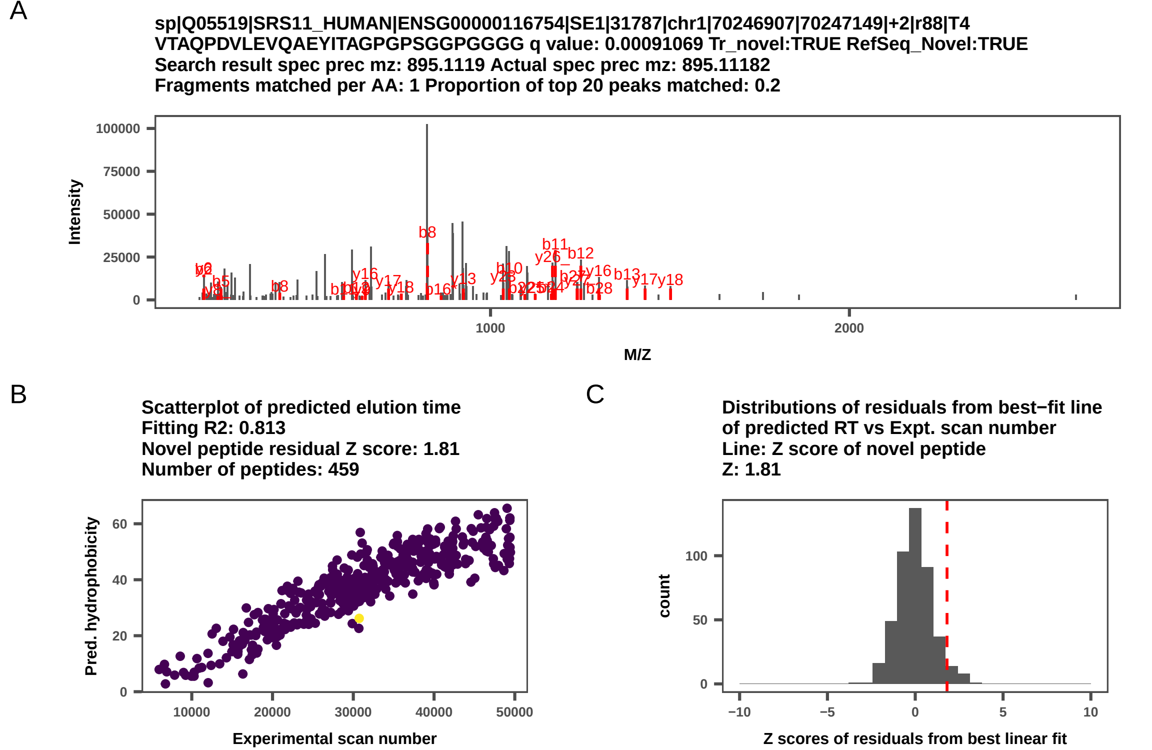
A
sp|Q05519|SRS11_HUMAN|ENSG00000116754|SE1|31787|chr1|70246907|70247149|+2|r88|T4
VTAQPDVLEVQAEYITAGPGPSGGPGGGG q value: 0.00091069 Tr_novel:TRUE RefSeq_Novel:TRUE
Search result spec prec mz: 895.1119 Actual spec prec mz: 895.11182
Fragments matched per AA: 1 Proportion of top 20 peaks matched: 0.2
Intensity
100000
75000
50000
25000
0
1000
2000
M/Z
b8
b11
b12
y26
b10
y16
y17
y18
y13
b13
b16
b28
b5
B
Scatterplot of predicted elution time
Fitting R2: 0.813
Novel peptide residual Z score: 1.81
Number of peptides: 459
Pred. hydrophobicity
Experimental scan number
10000
20000
30000
40000
50000
60
40
20
0
C
Distributions of residuals from best−fit line
of predicted RT vs Expt. scan number
Line: Z score of novel peptide
Z: 1.81
count
100
50
0
−10
−5
0
5
10
Z scores of residuals from best linear fit
